# Prolonged Doppler US-guided pneumatic compression of a radial artery pseudoaneurysm after percutaneous coronary intervention: a simple and effective solution for a rare and challenging problem

**DOI:** 10.1590/1677-5449.202102122

**Published:** 2023-10-13

**Authors:** Marcos Danillo Oliveira, Pedro Perillo, Lélio Lemos, Adriano Caixeta

**Affiliations:** 1 Universidade Federal de São Paulo - UNIFESP, Escola Paulista de Medicina, Hospital Universitário I, São Paulo, SP, Brasil.

**Keywords:** transradial access, complications, pseudoaneurysm, management, pneumatic compression, acesso arterial transradial, complicações, pseudoaneurisma, manejo, compressão pneumática

## Abstract

Transradial access is associated with fewer access site-related complications, earlier patient mobilization, and greater postprocedural comfort. Pseudoaneurysms are an extremely rare complication after transradial procedures and the radial artery itself is the most atypical arterial site of occurrence. We report a case in which a non-surgical, non-invasive, simple, and effective solution (prolonged pneumatic compression) was used to manage a radial artery pseudoaneurysm, a very rare and challenging complication of transradial procedures.

## Resumo

O acesso arterial transradial está associado a menos complicações relacionadas ao sítio de punção, com deambulação precoce do paciente e maior conforto pós-procedimento. O pseudoaneurisma é uma complicação extremamente rara após procedimentos transradiais, sendo a artéria radial, por si só, o sítio mais incomum para tal ocorrência. Relata-se um caso de um pseudoaneurisma de artéria radial, uma complicação rara e desafiadora, resolvido com êxito e de maneira simples, não invasiva e não cirúrgica (compressão pneumática prolongada).

## INTRODUCTION

Compared with the classic transfemoral access, transradial access has been shown to be cost-effective, with fewer access site-related complications, earlier patient mobilization, and greater postprocedural comfort. Although uncommon, it is still associated with some vascular complications: spasm, thrombotic occlusion, hematoma, pseudoaneurysm, arteriovenous fistula, and compartment syndrome.^1^ We report herein a case in which a non-surgical, non-invasive, simple, and effective solution was used for management of a radial artery pseudoaneurysm, a very rare and challenging complication of transradial procedures.

The Research Ethics Committee approved this study (decision number 4.071.731).

## CASE DESCRIPTION

**Figure 1:**
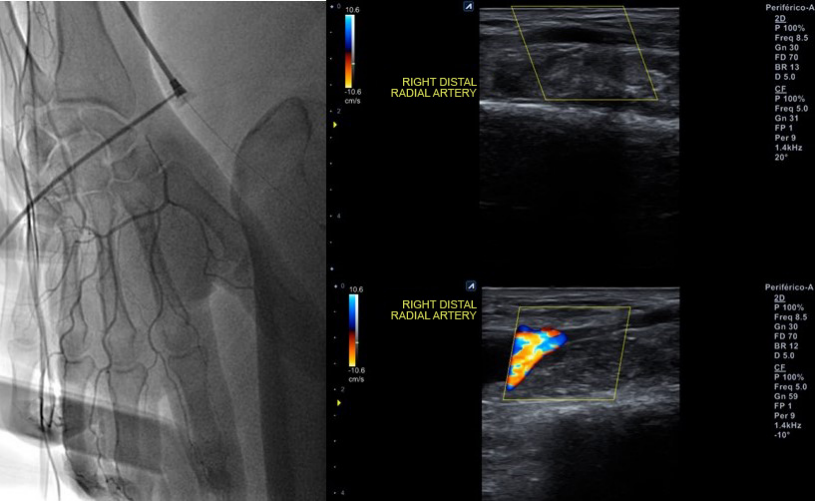
Right distal radial artery occlusion by angiography via proximal transradial sheath side port (left panel) and by post-procedure Doppler ultrasound (right panel).

**Figure 2:**
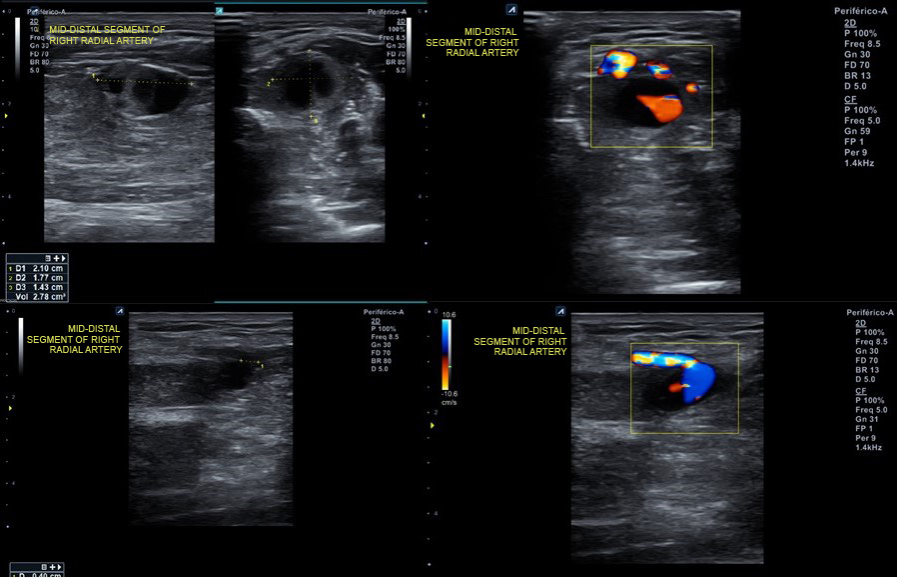
Post-procedure Doppler ultrasound evidenced proximal radial artery pseudoaneurysm.

**Figure 3:**
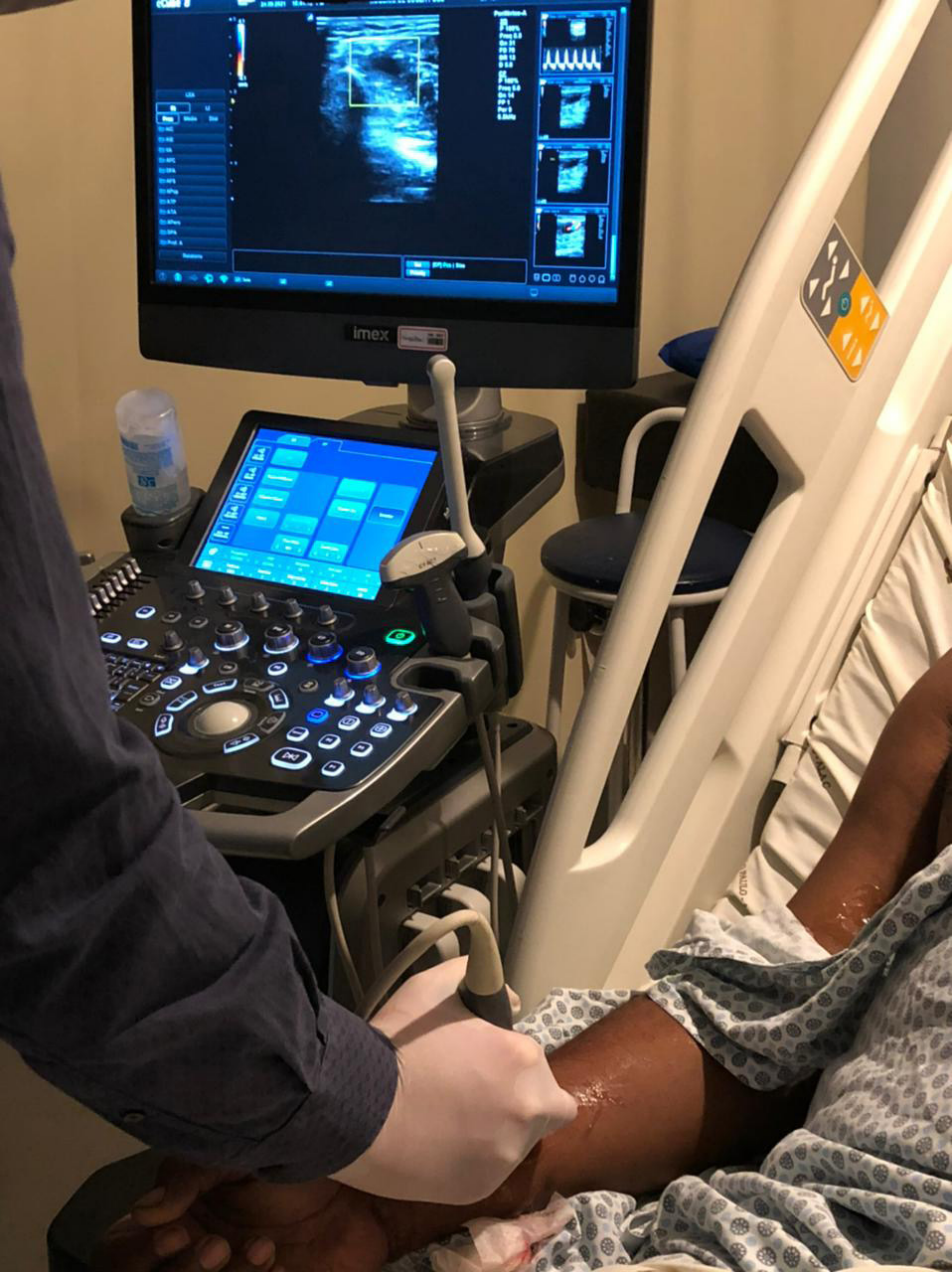
Doppler ultrasound-guided compression of pseudoaneurysm neck with probe.

**Figure 4:**
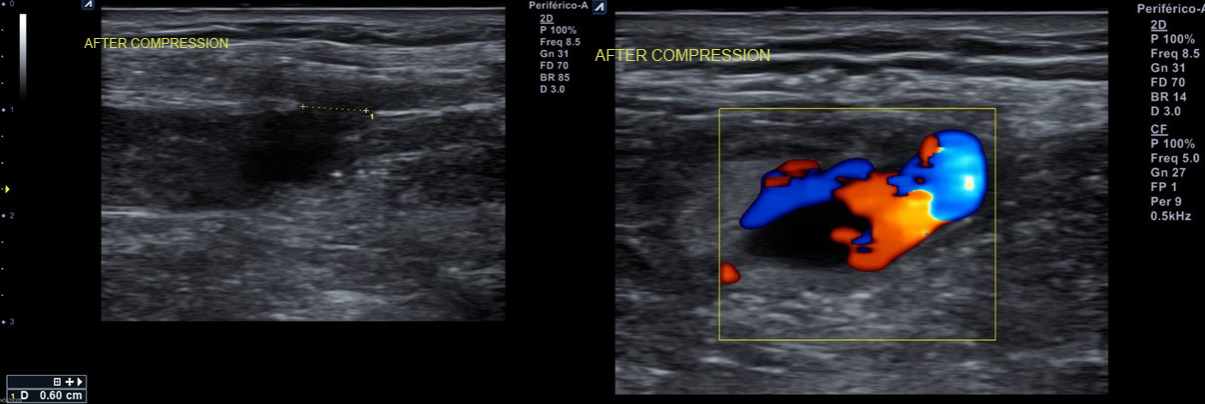
Doppler ultrasound after unsuccessful probe compression.

**Figure 5:**
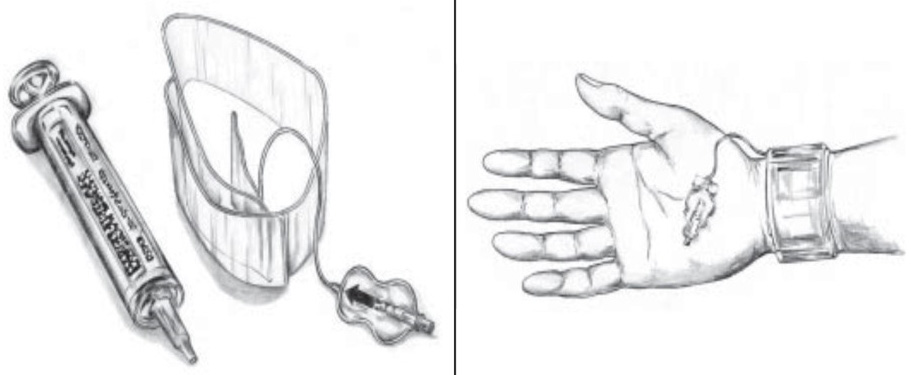
Hemostasis with the TR BAND™ Radial Compression Device (Terumo Corp., Tokyo, Japan).

**Figure 6:**
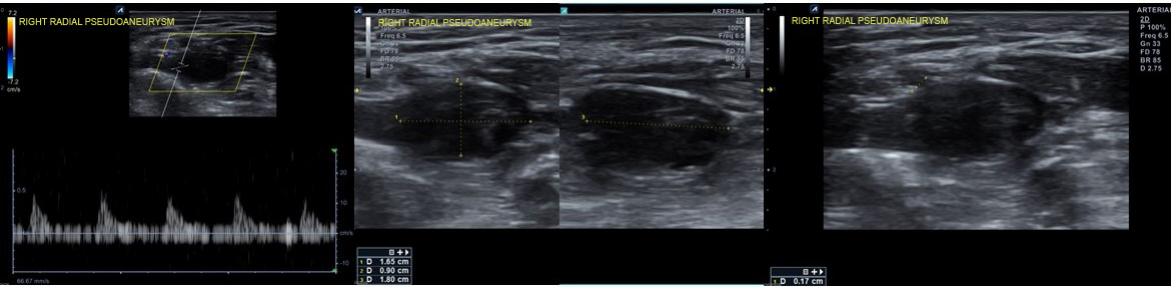
Doppler ultrasound confirmed thrombotic pseudoaneurysm occlusion after prolonged pneumatic neck compression.

**Figure 7:**
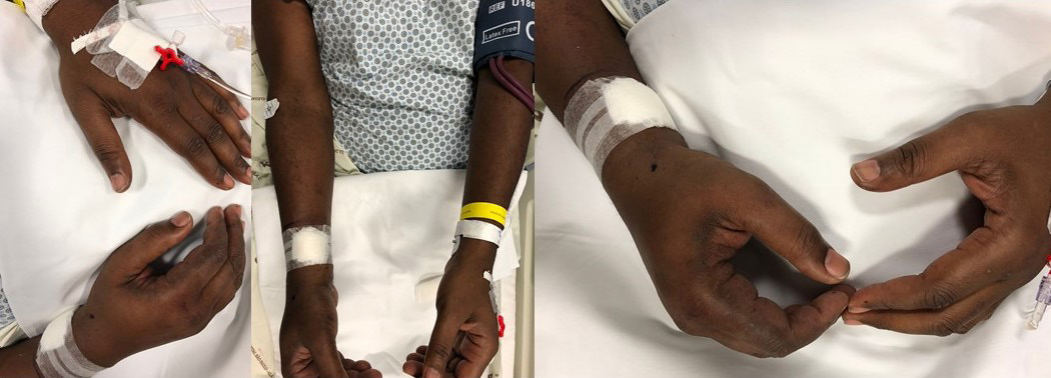
Physical appearance after prolonged pneumatic compression.

**Figure 8:**
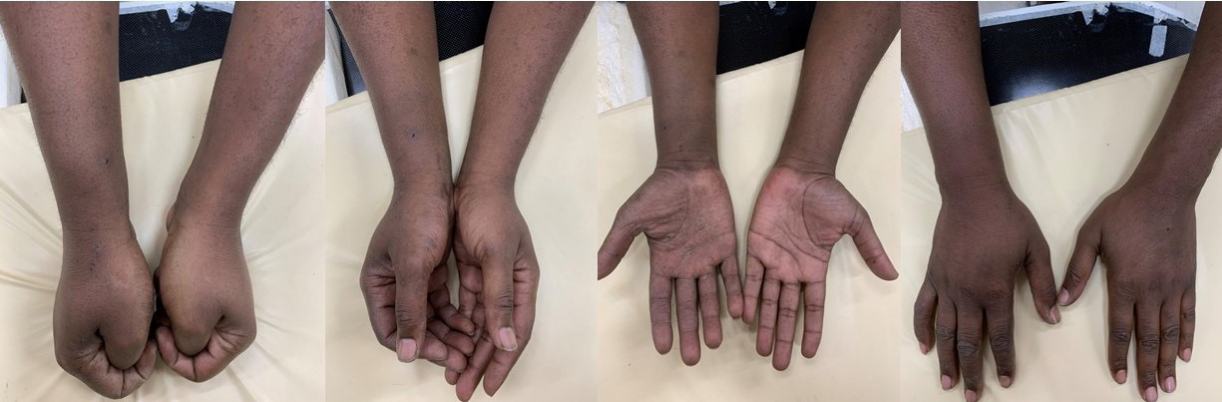
Physical appearance at seven-day follow-up.

**Figure 9:**
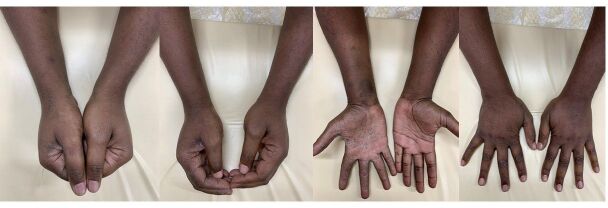
Physical appearance at forty-day follow-up.

**Figure 10:**
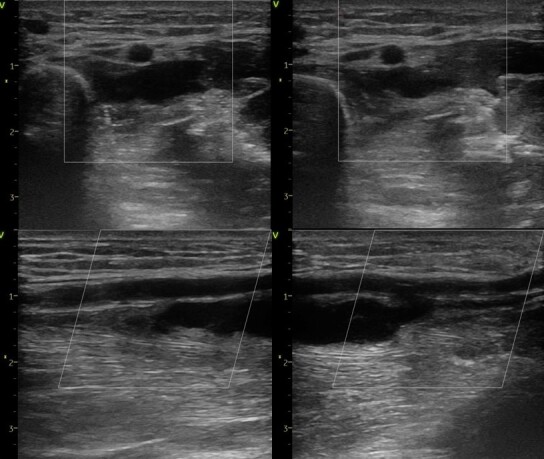
Doppler ultrasound confirmation of successful thrombotic pseudoaneurysm occlusion at forty-day follow-up.

A 50-year-old man with hypertension, diabetes, dyslipidemia, obesity, and an active smoking habit was referred to the cath lab due to stable angina in response to any minimal effort. Elective coronary angiography was uneventfully performed via right distal transradial access 6Fr, our default access site for routine coronary angiography and interventions.^2-6^ Severe ostial stenosis of the right posterior descending artery was found and elective percutaneous coronary intervention (PCI) was planned. A handmade hemostatic gauze pad was left in situ for 30 minutes and completely removed after one hour, without bleeding. Proximal and distal radial artery (RA) pulses were palpable after hemostasis and at hospital discharge, 4h later, which was uneventful. One week later, the patient was brought back to the cath lab for the planned PCI. Despite palpable right distal RA pulse, it was not possible, after five successful punctures, to advance even a polymer-jacketed 0.0014″ guidewire, probably due to distal RA thrombotic occlusion. It was decided to convert to proximal ipsilateral RA access. After prompt successful puncture, a 0.0014″ guidewire was easily advanced up to the aortic root. Sudden, progressive, and painful forearm swelling developed, so RA perforation was suspected. Due to previous successful experiences with spontaneous sealing after sheath and catheter insertion,^7^ this strategy was adopted. During hydrophilic 6Fr sheath advancement, guidewire looping was noticed and easily unfolded (Supplementary Material Video 1) with wire manipulation and sheath advancement. The PCI was performed as planned. An angiogram during guiding catheter removal showed no evidence of any proximal radial artery perforation or any extravascular dye staining. A final sheath angiography confirmed right distal RA occlusion (Figure 1; Supplementary Material Video 2). Brachial, ulnar, and radial pulses were palpable, distal perfusion was preserved to all fingers, and the forearm swelling diminished. Proximal radial artery patent hemostasis was performed with a hemostatic device. After removal, there was recrudescence of the painful forearm swelling. Doppler ultrasound (US) evidenced a 2.1 x 1.8 x 1.4cm (2.78cm^3^) pseudoaneurysm (Figure 2) with a 0.4cm neck. After continuous 30 min US-guided compression of the pseudoaneurysm neck (Figure 3), complete thrombotic occlusion was not achieved (Figure 4). Following precise neck location, a hemostatic device (TR BAND™ Radial Compression Device (Terumo Corp., Tokyo, Japan) was placed and inflated with 20mL of air (Figure 5), with maintenance of antegrade flow though the RA shown by Doppler US. After 4 hours uninterrupted compression, Doppler US showed proximal RA patency and thrombotic occlusion of the pseudoaneurysm (Figure 6). Physical examination (Figures 7, 8, and 9) and Doppler US follow-up (Figure 10) up to forty days after the index procedure corroborated complete resolution of the RA pseudoaneurysm.

## DISCUSSION

Pseudoaneurysm is an extremely rare complication after transradial procedures, with a rate of occurrence of about 0.05%,^8-9^ while the RA itself is the most atypical arterial site of pseudoaneurysm occurrence.^10^ It is the result of inadequate thrombus formation after catheter/sheath removal, causing a hematoma that communicates with the arterial lumen. Risk factors are repeated arterial punctures, catheter infection, advanced age, longer procedural duration, coagulation disorders or anticoagulants/antiplatelet agents, large sheath diameter, and incomplete hemostasis.^9-12^

The aim of radial pseudoaneurysm management is to repair the wall lesion and/or discontinue the flow communication between the artery and the hematoma.^10^ US-guided compression until occlusion is achieved can be performed and US-guided thrombin injection can also be undertaken, but fewer successful case reports exist for the RA.^13-16^ In addition, thrombin injection into the RA poses a significant risk of distal embolization and digital ischemia. This treatment has not been seriously considered. Surgical management is recommended in patients with large pseudoaneurysms, or those that are symptomatic, expanding, infected, subacute, or when initial conservative management has failed.^17^

Oliveira et al.^18^ and Prejean et al.^19^ reported successful management of pseudoaneurysms after distal transradial access by prolonged Doppler US-guided neck compression with pneumatic devices. In both cases, thrombosis of the pseudoaneurysm was achieved and no further intervention was required, exactly like in the present case.

Of note, an extensive literature review found no descriptions regarding the success rates of either of the aforementioned strategies or any specific relationships with ultrasonographic pseudoaneurysm features.

In conclusion, the present report suggests that, in this particular case, prolonged pneumatic compression appeared to be promising for pseudoaneurysm management. Further prospective studies are needed in order to assure its efficacy and safety. Also, in case of acute thrombotic occlusion after distal transradial procedures, proximal ipsilateral transradial access is still a feasible option for future interventions.

## Supplementary Material

Supplementary material accompanies this paper.

Video 1 During hydrophilic 6Fr sheath advancement, guidewire looping was noted (inside the pseudoaneurysm cavity) and easily unfolded with wire manipulation and sheath advancement.

Video 2 Right distal radial artery occlusion by angiography via right proximal transradial sheath side port.

This material is available as part of the online article from https://doi.org/10.1590/1677-5449.202102122
